# Antenatal care and uptake of HIV testing among pregnant women in sub-Saharan Africa: a cross-sectional study

**DOI:** 10.7448/IAS.19.1.20605

**Published:** 2016-01-18

**Authors:** Jayleen K L Gunn, Ibitola O Asaolu, Katherine E Center, Steven J Gibson, Patrick Wightman, Echezona E Ezeanolue, John E Ehiri

**Affiliations:** 1 Department of Epidemiology and Biostatistics, Mel and Enid Zuckerman College of Public Health, University of Arizona, Tucson, AZ, USA; 2 Department of Health Promotion Sciences, Mel and Enid Zuckerman College of Public Health, University of Arizona, Tucson, AZ, USA; 3 Department of Obstetrics and Gynecology, University of Arizona, Tucson, AZ, USA; 4 University of Arizona Cancer Center, Tucson, AZ, USA; 5 Center for Population Health, Arizona Health Sciences Center (AHSC), Center for Population Science and Discovery, College of Medicine, University of Arizona, Tucson, AZ, USA; 6 Global Health and Implementation Research Initiative, Department of Environmental and Occupational Health, School of Community Health Sciences, University of Nevada Las Vegas, Las Vegas, NV, USA

**Keywords:** Antenatal care, prenatal care, HIV testing, PMTCT, MTCT, maternal and child health, low-income countries, sub-Saharan Africa, global health, global maternal and child health

## Abstract

**Introduction:**

Current guidelines recommend inclusion of HIV testing in routine screening tests for all pregnant women. For this reason, antenatal care (ANC) represents a vital component of efforts to prevent mother-to-child transmission (PMTCT) of HIV. To elucidate the relationship between ANC services and HIV testing among pregnant women in sub-Saharan Africa, we undertook an analysis of data from four countries.

**Methods:**

Four countries (Congo, Mozambique, Nigeria and Uganda) were purposively selected to represent unique geographical regions of sub-Saharan Africa. Using Demographic and Health Survey datasets, weighted crude and adjusted logistic regression models were used to explore factors that influenced HIV testing as part of ANC services. The study was approved by the Institutional Review Board of the University of Arizona.

**Results:**

Pooled results showed that 60.7% of women received HIV testing as part of ANC. Ugandan women had the highest rate of HIV testing as part of ANC (81.5%) compared with women in Mozambique (69.4%), Nigeria (54.4%) and Congo (45.4%). Difficulty reaching a health facility was a barrier in Congo and Mozambique but not Nigeria or Uganda. HIV testing rates were lower in rural areas, among the poorest women, the least educated and those with limited knowledge of HIV. In every country, crude regression analyses showed higher odds of being tested for HIV if women received their ANC services from a skilled attendant compared with an unskilled attendant. After adjusting for confounders, women in the total sample had 1.78 (99% CI: 1.45–2.18) times the odds of having an HIV test as part of their ANC if they went to a skilled attendant compared with an unskilled attendant.

**Conclusions:**

There is a need for integration of HIV testing into routine ANC service to increase opportunities for PMTCT programmes to reach HIV-positive pregnant women. Attention should be paid to the expansion of outreach services for women in rural settings, and to the training, supervision and integration of unskilled attendants into formal maternal and child health programmes. Education of pregnant women and their communities is needed to increase HIV knowledge and reduce HIV stigma.

## Introduction

Following the implementation of the 2003 “United States Leadership Against AIDS, Tuberculosis, and Malaria Act (P.L. 108-25)” that authorized the President's Emergency Plan for AIDS Relief (PEPFAR) [1], the use of antiretroviral therapy (ART) in high HIV burden countries was scaled-up and HIV-related deaths have significantly declined over the past decade. The Joint United Nations Programme on HIV/AIDS (UNAIDS) estimates that 9 million life-years have been saved in sub-Saharan Africa alone as a result of wider availability of ART [2,3]. Nonetheless, sub-Saharan Africa remains the region with the largest proportion of pregnant women living with HIV, accounting for nearly 85% of the global burden [4]. Historically, HIV testing was primarily delivered via voluntary counselling and testing that emphasized client-initiated testing. Numerous studies reported the inadequacies of this model [5,6], leading the World Health Organization (WHO) to establish guidelines in 2004 that recommended routine HIV testing by healthcare providers for all pregnancy-related visits, especially in high HIV transmission areas [7]. As a result of the implementation of this approach in sub-Saharan Africa, HIV diagnoses in women are most often made during pregnancy [8].

Knowledge of HIV status allows pregnant women access to prevention of mother-to-child transmission (PMTCT) services [9]. PMTCT services are designed to reduce the risk of HIV transmission from a HIV-positive mother to her child [9]. Mother-to-child transmission (MTCT) – also known as vertical transmission – is the most common cause of paediatric HIV infection, often occurring during pregnancy, birth or breastfeeding [10]. In 2013, the WHO recommended two approaches to PMTCT for HIV-positive pregnant women: Option B+ or, if this is not possible, Option B [4]. In Option B+, all HIV-positive pregnant women are placed on ART for life, regardless of their CD4 count or WHO clinical stage [4]. In Option B, pregnant women with HIV who are eligible for ART (CD4 counts under 500 or clinical stage 3 or 4) should be treated with ART for life; however, those who are not eligible for ART should stop ART after delivery or a week after completion of breastfeeding [4]. The PMTCT guidelines also recommend optimal breastfeeding techniques for HIV-positive mothers living in developing countries: exclusive breastfeeding for the first 6 months of life, followed by continued breastfeeding combined with nutritionally adequate complementary feeding up to 24 months of age. Furthermore, since 2010, the PMTCT guidelines have recommended exclusive formula feeding for women for whom it is acceptable, feasible, affordable, sustainable and safe [11].

While uptake of PMTCT services in sub-Saharan Africa has increased from 3% in 2003 to 73% in 2014, there remains a substantial need to increase PMTCT interventions [5,12,13] due to their direct effects on reduction of vertical transmission of HIV [14,15]. Antenatal care (ANC) provides an opportunity for integration of routine maternal and child health (MCH) services with HIV screening and thus represents a critical entry point into the PMTCT care cascade [16–18]. Service integration has the potential to improve care and reduce missed opportunities for key interventions such as HIV testing, provision of ART, education in optimal breastfeeding and adherence support [19]. In fact, studies conducted in sub-Saharan Africa have demonstrated an association between service integration – delivery of services or multiple interventions together at the same patient visit by the same health worker or clinical team – and increased maternal HIV care enrolment and uptake of ART [16–18]. However, in sub-Saharan Africa, uptake of both overall medical care and ANC is sub-optimal. Thus, it is important that women who do access ANC are provided all of the recommended medical tests (including HIV tests) and linked to appropriate care through effective service integration.

Missed opportunities for HIV screening of pregnant women are perilous; knowing a woman's HIV status and enrolling her into available PMTCT services can help to reduce vertical HIV transmission [20]. Understanding and addressing barriers against integration of ANC and HIV testing and care could significantly contribute to the current global goal of eliminating MTCT of HIV [20]. The aim of this study was to assess individual and health system–level factors that influence HIV testing as part of ANC service in four countries in sub-Saharan Africa.

## Methods

### Survey

Data were obtained from the Demographic and Health Survey (DHS). For this study, DHS datasets were individually analyzed for each country. Four countries that represent unique geographical regions of sub-Saharan Africa were purposively selected: Congo (Brazzaville), representing central Africa (DHS 2011–2012); Mozambique, representing southern Africa (DHS 2011); Nigeria, representing western Africa (DHS 2013); and Uganda, representing eastern Africa (DHS 2011). Information regarding the collection and sampling techniques employed by DHS have been previously published in detail [21]. In brief, DHS used a cross-sectional survey design to obtain nationally representative data. The surveys were typically designed to collect information on demographics, family planning, HIV and other aspects of health. Data included a standard set of questions; however, some survey items reflect country-specific issues. Only women who received ANC during their last pregnancy – which was within 5 years preceding the survey – were retained for the analyses in this paper.

### Measures

In the DHS, an indicator for comprehensive knowledge of HIV was created using UNICEF's definition of comprehensive knowledge [22]. Women were classified as having comprehensive HIV knowledge if they correctly: 1) identified two methods of preventing sexual transmission of HIV; 2) acknowledged that a healthy looking person can have HIV; and 3) rejected two common misconceptions about HIV transmission, that is, HIV can be transmitted through mosquito bites or by sharing food with a HIV-positive person [22]. A skilled ANC attendant was defined using the WHO's standard as an accredited health professional including a doctor, nurse or midwife [23]. Otherwise, the attendant was classified as non-skilled. Stigma towards HIV was defined as unwillingness to care for a relative with AIDS, purchase vegetables from someone known to be HIV-positive or allow a HIV-positive female teacher to continue teaching [24]. The DHS variable on difficulty in getting to a healthcare facility measured distance as a barrier to accessing a healthcare facility. Data were elicited using the item: “Getting medical help for self: distance to health facility” with corresponding response options as: “Big problem” versus “Not a big problem.”

### Statistical analysis

Due to oversampling of certain populations, individual weights were used as recommended by DHS [25]. Using weights allows for adjustment for non-response to questions and makes the data more representative of the study population on a national level. Weighted descriptive statistics were generated to demonstrate the sampling weight of each variable. Weighted chi-square tests were used to assess the relationship between pregnant women's characteristics and uptake of HIV testing. Weights were applied in all regression models. Using weighted logistic regression, the relationship between ANC provider (skilled or non-skilled attendant) and uptake of HIV testing was adjusted by participants’ characteristics (age, education, area of residence, difficulty accessing a healthcare facility, wealth index, stigma towards someone with HIV and comprehensive HIV knowledge). Statistical significance was set at *p*<0.01. All data cleaning, variable manipulation and analyses were conducted using SAS 9.4 (Cary, North Carolina) using the “PROC SURVEY” command. DHS surveys were conducted under the scientific and administrative oversight of the local country, including ethical review by the corresponding local ethics review board. Data collection procedures were also approved by the ORC Macro institutional review board. In addition, this secondary data analysis was evaluated by the Mel and Enid Zuckerman College of Public Health Research Office and was considered exempt from human subjects review.

## Results

Of the 25,201 pregnant women included in the analyses, 14.6% were Congolese, 24.8% were Mozambicans, 46.2% were Nigerians and 14.3% were Ugandans.

### Congo

Table 1 presents a full description of characteristics of the Congolese participants. Briefly, 53.1% of the sampled women were aged 20–29, 62.8% had secondary school education, 67.6% lived in an urban environment and 39.2% of the women reported difficulty getting to a health care facility. The women were generally evenly distributed along the wealth index: poorest (16.5%), poorer (22.4%), middle (22.0%), richer (20.8%) and richest (18.3%). Over half of the sample (52.1%) had a stigma towards someone with HIV, whereas less than half (38.9%) had comprehensive HIV knowledge. Most women in Congo (96.9%) saw a skilled attendant for ANC; however, less than half (45.5%) had been tested for HIV during that time.

**Table 1:** Participant characteristics by country and total, using the DHS weighted sampling unit

	Congo		Mozambique		Nigeria		Uganda		Total	
					
	*N*	%	*N*	%	*N*	%	*N*	%	*N*	%
Age										
15–19	439	11.8	786	12.4	556	4.7	329	9.0	2109	8.3
20–29	1974	53.1	3111	49.3	5353	45.5	2046	56.0	12,484	49.0
30–39	1139	30.6	1973	31.2	4592	39.1	1073	29.4	8777	34.5
40–49	169	4.5	446	7.1	1265	10.7	203	5.6	2082	8.2
Education										
None	174	4.7	2107	33.4	3104	26.3	482	13.2	5867	23.1
Primary	1046	28.1	3324	52.6	2702	23.0	2297	62.9	9369	36.8
Secondary	2336	62.8	842	13.3	4726	40.2	730	20.0	8635	33.9
Tertiary	164	4.4	43	0.7	1234	10.5	142	3.9	1582	6.2
Residence										
Urban	2515	67.6	1919	30.4	6135	52.1	548	15.0	11,117	43.7
Rural	1205	32.4	4397	69.6	5631	47.9	3103	85.0	14,336	56.3
Difficulty accessing healthcare facility										
Yes	1457	39.2	3508	55.5	2434	20.7	1656	45.4	8838	35.6
No	2263	60.8	2808	44.5	9332	79.3	1994	54.6	16,363	64.4
Wealth index										
Poorest	613	16.5	1302	20.6	1018	8.7	801	22.0	3735	14.7
Poorer	835	22.4	1308	20.7	1871	15.9	769	21.0	4784	18.8
Middle	820	22.0	1252	19.8	2448	20.8	715	19.6	5235	20.6
Richer	772	20.8	1341	21.2	3039	25.8	662	18.1	5814	22.8
Richest	679	18.3	1113	17.7	3389	28.8	704	19.3	5885	23.1
HIV stigma										
Yes	1938	52.1	2333	36.9	7306	62.1	1613	44.2	13,191	51.8
No	1782	47.9	3983	63.1	4460	37.9	2037	55.8	12,262	48.2
HIV knowledge										
Yes	1448	38.9	2028	32.1	4422	37.6	1297	35.5	9195	36.1
No	2272	61.1	4288	67.9	7344	62.4	2354	64.5	16,257	63.9
ANC attendant										
Skilled	3605	96.9	4125	65.3	11,100	94.3	3559	97.5	22,389	88.0
Unskilled	115	3.1	2192	34.7	666	5.7	91	2.5	3064	12.0
HIV tested during ANC										
Yes	1693	45.5	4386	69.4	6395	54.4	2976	81.5	15,451	60.7
No	2027	54.5	1930	30.6	5371	45.6	674	18.5	10,002	39.3

Note: Each *N* and percentage represents a weighted number calculated by using the DHS weighted sampling unit and PROC SURVEY command in SAS.

Table 2 shows the relationship between HIV testing during ANC and participants’ characteristics. There was a significant association between being tested for HIV as part of ANC and education (*p<*0.0001); those with a tertiary education had the greatest uptake of HIV testing (71.9%) compared with those with secondary, primary or no education (50.1, 34.7 and 23.7%, respectively). There was also a statistically significant association between area of residence and receipt of HIV test as part of ANC (*p<*0.0001), with more women who reside in urban areas (51.6%) being tested for HIV during ANC compared with women who lived in rural communities (32.8%). Difficulty accessing a healthcare facility was also significantly associated with uptake of HIV testing (*p*=0.004); only 40.2% of women who reported difficulty accessing a healthcare facility had an HIV test during ANC compared with 49.0% of women who had no such difficulty. Wealth index was also significantly associated with uptake of HIV during ANC testing (*p<*0.0001). As women's wealth quintile increased, so too did the percentage of women who partook in HIV testing as part of ANC (poorest, 24.4%; poorer, 34.5%; middle, 44.5%; richer, 57.8%; and richest, 65.4%). Women who expressed stigma towards someone with HIV were less likely to have an HIV test as part of ANC (37.0%) than those who did not express stigma (54.8%, *p<*0.0001). Comprehensive HIV knowledge was associated with uptake of HIV testing as part of ANC (*p*=0.0016); 50.5% of women with comprehensive HIV knowledge were tested for HIV as part of their ANC compared with 42.3% of women who lacked comprehensive HIV knowledge. Finally, more women who received ANC from a skilled attendant were tested for HIV test as part of ANC (46.1%) compared with those who received ANC from an unskilled attendant (25.8%, *p*=0.0003). No relationship was observed between participants’ age and uptake of HIV test as part of ANC (*p*=0.1591).

**Table 2:** Chi-square testing the relationship between uptake of HIV testing during ANC and participant characteristics

	Congo			Mozambique			Nigeria			Uganda			Total		
					
	Not tested, %	Tested, %	*p*	Not tested, %	Tested, %	*p*	Not tested, %	Tested, %	*p*	Not tested, %	Tested, %	*p*	Not tested, %	Tested, %	*p*
Age															
15–19	59.6	40.4	0.1591	31.6	68.4	<0.0001	63.0	37.0	<0.0001	16.2	83.8	0.0002	43.3	56.7	<0.0001
20–29	52.5	47.5		27.5	72.5		46.4	53.6		16.6	83.4		37.8	62.2	
30–39	55.0	45.0		32.5	67.5		41.6	58.4		20.7	79.3		38.7	61.3	
40–49	61.4	38.6		41.4	58.6		49.3	50.7		29.0	71.0		46.6	53.4	
Education															
None	76.3	23.7	<0.0001	41.8	58.2	0.0001	70.0	30.0	<0.0001	31.7	68.3	<0.0001	56.9	43.1	<0.0001
Primary	65.3	34.7		30.1	69.9		55.6	44.4		18.5	81.5		38.5	61.5	
Secondary	49.9	50.1		5.9	94.1		33.0	67.0		11.9	88.1		33.1	66.9	
Tertiary	28.1	71.9		1.3	98.7		10.9	89.1		6.7	93.3		12.0	88.0	
Residence															
Urban	48.4	51.6	<0.0001	17.1	82.9	<0.0001	33.1	66.9	<0.0001	7.3	92.7	<0.0001	32.5	67.5	<0.0001
Rural	67.2	32.8		36.4	63.6		59.3	40.7		20.4	79.6		44.5	55.5	
Difficulty accessing healthcare facility															
No	51.0	49.0	0.0040	21.2	78.8	<0.0001	42.1	57.9	<0.0001	16.7	83.3	0.0061	36.7	63.3	<0.0001
Yes	59.8	40.2		38.0	62.0		59.3	40.7		20.6	79.4		44.1	55.9	
Wealth index															
Poorest	75.6	24.4	<0.0001	46.9	53.1	<0.0001	79.0	30.0	<0.0001	24.2	75.8	<0.0001	55.5	44.5	<0.0001
Poorer	65.5	34.5		42.0	58.0		67.9	32.1		22.5	77.5		53.1	46.9	
Middle	55.5	44.5		33.4	66.6		53.5	46.5		17.9	82.1		44.1	55.9	
Richer	42.2	57.8		20.3	79.7		40.4	59.6		16.6	83.4		33.3	66.7	
Richest	34.6	65.4		7.0	93.0		22.4	77.6		9.9	90.1		19.4	80.6	
HIV stigma															
No	45.2	54.8	<0.0001	24.7	75.3	<0.0001	34.7	65.3	<0.0001	14.2	85.8	<0.0001	29.6	70.4	<0.0001
Yes	63.0	37.0		40.6	59.4		52.3	47.7		23.8	76.2		48.3	51.7	
HIV knowledge															
No	57.7	42.3	0.0016	33.3	66.7	<0.0001	52.4	47.6	<0.0001	20.7	79.3	0.0001	43.5	56.5	<0.0001
Yes	49.5	50.5		24.7	75.3		34.4	65.6		14.3	85.7		31.8	68.2	
ANC attendant															
Skilled	53.9	46.1	0.0003	24.3	75.7	<0.0001	44.3	55.7	<0.0001	17.6	82.4	<0.0001	37.9	62.1	<0.0001
Unskilled	74.2	25.8		42.2	57.8		68.5	31.5		50.6	49.4		49.4	50.6	

*p*<0.01 significant.

Note: Each chi-square represents a weighted calculation by using the DHS weighted sampling unit and PROC SURVEY command in SAS.

Table 3 shows outcome of crude and adjusted logistic regression models for the relationship between having an HIV test and whether the ANC attendant was skilled or unskilled. The crude models showed an increase in the odds of women who saw a skilled ANC attendant having an HIV test as part of ANC compared with those who saw an unskilled attendant for their ANC (OR: 2.46; 99% CI: 1.26–4.81). However, when adjusting for potential confounders, no association between skilled or unskilled attendant and uptake of HIV testing was observed (Adjusted OR [aOR]: 1.43; 99% CI: 0.79–2.59).

**Table 3:** Logistic regression testing uptake of HIV testing during ANC by skilled versus unskilled ANC attendant

	Congo	Mozambique	Nigeria	Uganda	Total^a^
Crude					
Skilled Attendant^b^	2.46 (1.26–4.81)*	2.27 (1.78–2.90)*	2.74 (1.80–4.16)*	4.79 (2.61–8.81)*	1.60 (1.32–1.94)*
Unskilled Attendant^c^	*Ref*.	*Ref*.	*Ref*.	*Ref*.	*Ref*.
Adjusted^d^,^e^					
Skilled Attendant^b^	1.43 (0.79–2.59)	1.58 (1.22–2.05)*	1.95 (1.30–2.93)*	4.48 (2.48–8.08)*	1.78 (1.45–2.18)*
Unskilled Attendant^c^	*Ref*.	*Ref*.	*Ref*.	*Ref*.	*Ref*.

* Signifies significant relationship at *a*=0.01.

a Adjusted by country.

b Skilled attendant defined as doctor, nurse or midwife.

c Unskilled attendant defined as auxiliary midwife, community/village health worker or traditional birth attendant.

d Weighted N for adjusted regression: 3720.0 Congo; 6316.3 Mozambique; 11,765.9 Nigeria; 3650.6 Uganda; 25,452.7 Total.

e Models adjusted for maternal age, education, residence, difficulty accessing a healthcare facility, wealth index, stigma towards someone with HIV and comprehensive knowledge of HIV.

### Mozambique

Table 1 presents the characteristics of respondents from Mozambique. Briefly, most women were aged 20–29 years (49.3%), had a primary education (52.6%), lived in a rural setting (69.6%) and reported difficulty getting to a healthcare facility (55.5%). The women from Mozambique were also generally evenly distributed along the wealth index: poorest (20.6%), poorer (20.7%), middle (19.8%), richer (21.2%) and richest (17.7%). Less than half of the sample (36.9%) reported stigma towards women with HIV; however, only 32.1% had comprehensive HIV knowledge. More women (65.3%) received ANC from skilled attendants than from unskilled attendants (34.7%). Finally, more than two-thirds of women (69.4%) were tested for HIV as part of their ANC.

Table 2 shows the relationship between participants’ characteristics and receipt of HIV testing as part of ANC. There was a significant association between age and HIV testing as part of ANC (*p<*0.0001), with more women aged 20–29 years having an HIV test as part of ANC (72.5%) compared with those 15–19 years (68.4%), 30–39 years (67.5%) and 40–49 years (58.6%). Education was significantly associated with having an HIV test as part of ANC (*p<*0.0001). As education increased so too did the per cent of women who partook in HIV testing as part of ANC (no education, 58.2%; primary, 69.9%; secondary, 94.1%; and tertiary education, 98.7%). Type of living environment was also significantly associated with having an HIV test as part of ANC (*p<*0.0001), with more women in urban locations having an HIV test as part of ANC (82.9%) compared with those in rural locations (63.6%). Women who reported difficulty accessing a healthcare facility were less likely to be tested for HIV as part of ANC compared with those who did not report having difficulty accessing a healthcare facility (62.0 and 78.8%, respectively; chi-square *p<*0.0001). Wealth index was also significantly related to having an HIV test as part of ANC (*p<*0.0001). As women's wealth quintile increased so too did rates of having an HIV test (poorest, 53.1%; poorer, 58.0%; middle, 66.6%; richer, 79.7%; and richest, 93.0%). Fewer women had an HIV test as part of ANC if they displayed stigma towards someone with HIV (59.4%) compared with those who did not display stigma towards someone with HIV (75.3%; chi-square *p<*0.0001). Having comprehensive HIV knowledge was significantly associated with having an HIV test as part of ANC (*p<*0.0001); more women who displayed comprehensive HIV knowledge had an HIV test (75.3%) compared with those who lacked comprehensive HIV knowledge (66.7%). Finally, women who received ANC from a skilled attendant were more likely to have HIV tests during ANC (75.7%) compared with those who received ANC from an unskilled attendant (57.8%; chi-square *p<*0.0001). As shown in Table 3 for both crude and adjusted logistic regression models, an increase in the odds of having an HIV test during ANC was observed if the participant saw a skilled attendant for ANC compared with if they saw an unskilled attendant (aOR:1.58; 99% CI: 1.22–2.05).

### Nigeria

Table 1 presents a description of characteristics of Nigerian participants. Briefly, 45.5% of the women were aged 20–29 years, 40.2% had a secondary education, 52.1% lived in an urban setting and 20.7% reported difficulty accessing a healthcare facility. Women's wealth quintile was as follows: poorest (8.7%), poorer (15.9%), middle (20.8%), richer (25.8%) and richest (28.8%). Over half of the sample (62.1%) reported stigma towards someone with HIV, and few (37.6%) had comprehensive HIV knowledge. Almost all of the women (94.3%) received their ANC from a skilled attendant, but only 54.4% were tested for HIV during that time.

Table 2 shows the relationship between being tested for HIV and participants’ characteristics. There was a significant relationship between uptake of HIV testing and age (*p<*0.0001). More women aged 30–39 years (58.4%) were tested for HIV as part of ANC compared with those aged 15–19 (37.0%), 20–29 (53.6%) and 40–49 (50.7%). Education was significantly associated with having an HIV test as part of ANC (*p<*0.0001). As education increased, so did uptake of HIV testing as part of ANC (no education, 30.0%; primary, 44.4%; secondary, 67.0%; and tertiary education, 89.1%). Women who lived in urban areas were more likely to be tested for HIV as part of ANC compared with those who lived in rural areas (66.9 and 40.7%, respectively; chi-square *p<*0.0001). Women were less likely to be tested for HIV as part of ANC if they had difficulty accessing a healthcare facility (40.7%) compared with those who did not have difficulty accessing a healthcare facility during pregnancy (57.9%; chi-square *p<*0.0001). Wealth index was significantly associated with having an HIV test as part of ANC (*p<*0.0001). As women's wealth quintile increased, so did the per cent of women who received an HIV test during ANC (poorest, 30.0%; poorer, 32.1%; middle, 46.5%; richer, 59.6%; and richest, 77.6%). Women who displayed stigma towards someone with HIV were less likely to be tested for HIV during ANC compared with women who did not display stigma towards someone with HIV (47.7 and 65.3%, respectively; chi-square *p<*0.0001). Women who displayed comprehensive HIV knowledge were more likely to test for HIV as part of ANC compared with those who lacked comprehensive HIV knowledge (65.6 and 47.6%, respectively; chi-square *p<*0.0001). Finally, women who received ANC from a skilled attendant were more likely to have an HIV test (55.7%), compared with those who received ANC from an unskilled attendant (31.5%; chi-square *p<*0.0001).

As shown in Table 3, for both crude and adjusted logistic regression models, an increase in the odds of having an HIV test was observed if the participant saw a skilled attendant for ANC compared with if they saw an unskilled attendant (aOR: 1.95; 99% CI: 1.30–2.93).

### Uganda

Characteristics of participants from Ugandan respondents are shown in Table 1. Over half of the women sample (56.0%) were aged of 20–29 years and had a primary school education (62.9%). A majority of the women (85%) lived in a rural setting and less than half (45.4%) indicated that they had difficulty accessing a healthcare facility. Women were spread fairly evenly across the wealth index: poorest (22.0%), poorer (21.0%), middle (19.6%), richer (18.1%) and richest (19.3%). Less than half of the sample (44.2%) displayed stigma towards people with HIV; however, only 35.5% had comprehensive HIV knowledge. Almost all of the women received their ANC from a skilled attendant (97.5%) and were tested for HIV as part of their ANC (81.5%).

Table 2 shows the relationship between uptake of HIV testing as part of ANC and participants’ characteristics. There was a significant association between receiving HIV testing as part of ANC and age (*p*=0.0002) with more women aged 15–19 and 20–29 having a HIV test as part of ANC (83.8 and 83.4%, respectively) compared with those aged 30–39 (79.3%) and 40–49 (71.0%). Education was significantly associated with having an HIV test as part of ANC (*p<*0.0001). As education increased, so did the per cent of women who received HIV testing as part of ANC (no education, 68.3%; primary, 81.5%; secondary, 88.1%; and tertiary education, 93.3%). Area of residence was also significantly associated with having an HIV test as part of ANC (*p<*0.0001); more women in urban locations had an HIV test as part of ANC (92.7%), compared with those in rural locations (79.6%). Women who experienced difficulty accessing a healthcare facility were less likely to be tested for HIV as part of ANC compared with those who did not have such a difficulty (79.4 and 83.3%, respectively; chi-square *p=*0.0061). Wealth index was also significantly related to having an HIV test as part of ANC (<0.0001). As women's wealth quintile increased, so did the likelihood of having an HIV test (poorest, 75.8%; poorer, 77.5%; middle, 82.1%; richer, 83.4%; and richest, 90.1%). Fewer women received HIV tests if they displayed stigma towards someone with HIV (76.2%) compared with those who did not display stigma towards someone with HIV (85.8%; chi-square *p<*0.0001). Having comprehensive HIV knowledge was significantly associated with having an HIV test as part of ANC (*p<*0.0001), with more women having an HIV test if they displayed comprehensive knowledge (85.7%) compared with those who did not have comprehensive HIV knowledge (79.3%). If a skilled attendant was used for ANC, women were more likely to receive HIV tests (82.4%) compared with those who used unskilled attendants (49.4%; chi-square *p<*0.0001).

Table 3 shows the results of the logistic regression. For both the crude and the adjusted models, an increase in the odds of having an HIV test was observed if the participant saw a skilled attendant for ANC compared with if they saw an unskilled attendant (aOR: 4.48; 99% CI: 2.48–8.08).

### All countries

Descriptive characteristics of the sample are summarized in Table 1. In brief, there were more women aged 20–29 (49.0%) than any other age group: 36.8% had a primary school education, 56.3% lived in a rural setting and 35.6% stated that they had difficulty accessing a healthcare facility. Women's wealth index were as follows: poorest (14.7%), poorer (18.8%), middle (20.6%), richer (22.8%) and richest (23.1%). Roughly half of the sample (51.8%) reported stigma towards those with HIV while less than half of the sample (36.1%) had comprehensive HIV knowledge. Although a majority of the women (88.0%) received ANC from a skilled attendant, only 60.7% received HIV testing as part of their ANC.

Table 2 shows the relationship between HIV testing and the participants’ characteristics. There was a significant relationship between uptake of HIV testing and age (*p<*0.0001). More women aged 20–29 years (62.2%) received HIV testing as part of ANC compared with women aged 15–19 years (56.7%), 30–39 years (61.3%) and 40–49 years (53.4%). Only in Uganda was uptake of HIV testing highest among the youngest group (15–19 years, Table 2). For all countries overall, there was also a significant association between education and uptake of HIV testing during ANC (*p<*0.0001); those with a tertiary education (88.0%) had the greatest uptake of HIV testing compared with secondary, primary and no education (66.9, 61.5 and 43.1%, respectively). There was also a statistically significant association between living in a rural or urban location (*p<*0.0001), with more women who reside in urban areas (67.5%) being tested for HIV during ANC compared with women who lived in rural communities (55.5%). Difficulty accessing a healthcare facility was significantly associated with uptake of HIV testing (*p<*0.0001); fewer women reported receiving an HIV test during ANC if they had difficulty accessing a healthcare facility than if they indicated no such difficulty (55.9 and 63.3%, respectively). Wealth index was also significantly associated with uptake of HIV during ANC testing (*p<*0.0001). As women's wealth quintile increased, so did the percentage of women who received HIV testing as part of ANC (poorest, 44.5%; poorer, 46.9%; middle, 55.9%; richer, 66.7%; and richest, 80.6%). Having stigma towards people with HIV was also associated with uptake of HIV testing as part of ANC (*p<*0.0001) with significantly fewer women having an HIV test if they showed stigma towards someone with HIV (51.7%) than if they did not display stigma (70.4%). Lack of comprehensive HIV knowledge was also significantly associated with uptake of HIV as part of ANC (*p<*0.0001), with 68.2% of women who had comprehensive HIV knowledge being tested for HIV compared with 56.5% of women who lacked such knowledge. Whether the ANC provider was skilled or unskilled was also significantly associated with having an HIV test as part of ANC (*p<*0.0001), with more women having an HIV test if they saw a skilled attendant for ANC (62.1%) compared with those who saw an unskilled attendant (50.6%).

As shown in Table 3, in the adjusted logistic regression model, an increase in the odds of having an HIV test was observed if the participant saw a skilled attendant for ANC compared with if they saw an unskilled attendant (OR: 1.78; 99% CI: 1.45–2.18).

## Discussion

While mother-to-child transmission of HIV has almost been eliminated in many high-income countries, it remains an important source of new HIV infections in sub-Saharan African countries. According to the 2014 report of the Joint United Nations Programme on HIV/AIDS (UNAIDS), sub-Saharan Africa accounted for 87% of the 1.5 million pregnant women living with HIV and 91% of children living with HIV worldwide in 2013 [26]. In spite of continued effort and the availability of simple, relatively inexpensive and highly effective ART for PMTCT of HIV, 32% of the women in the aforementioned UNAIDS report did not receive ART for PMTCT resulting in an estimated 240,000 new infections in children [26]. The enormous burden that paediatric HIV infections places on families and the financial strain HIV exerts on health systems warrant careful attention to efforts to eliminate MTCT of HIV in resource-poor settings. In many sub-Saharan African countries, less than 40% of deliveries occur in hospitals [27]. Thus, there is a need to optimize opportunities for HIV testing of the few pregnant women who do access antenatal services. Identification of HIV-positive pregnant women through routine HIV screening is a critical step to initiate PMTCT interventions, which is why current guidelines recommend HIV screening be part of the routine panel of screening tests for all pregnant women [7]. ANC facilities represent a critical entry point into the PMTCT care cascade. It is hoped that this paper will help to inform policy and practice related to integration of HIV testing in routine ANC services.

Overall, results of our analyses indicate that although a majority of the women (88.0%) received ANC from a skilled attendant, only 60.7% received HIV testing as part of ANC. Ugandan women had the highest rates of HIV testing as part of ANC with 81.5% of women tested compared with only 69.4% of Mozambican women, 54.4% of Nigerian women and 45.5% of Congolese women. For the overall sample, and for each country excluding Nigeria, women aged 40–49 years were least likely to receive HIV testing as part of ANC. In Nigeria, women aged 20–29 years were least likely to receive HIV testing as part of ANC. These age differences may reflect a need for educating ANC providers that women of all ages and relationship statuses can contract HIV and should receive HIV testing. Difficulty accessing HIV testing remains a problem in each country – except Uganda – with much lower HIV testing rates in rural areas, among the poorest and the least educated. The high rates of HIV testing among women in Uganda where a significant proportion of the population resides in rural areas may be due to local policies that ensure higher HIV testing rates such as the opt-out testing during ANC and mandatory testing for people receiving care in public health centres [6,7]. HIV testing rates were higher among women who did not express stigmatizing attitudes towards persons living with HIV/AIDS and among those with comprehensive HIV knowledge. In every country, crude regression analyses showed higher odds of being tested for HIV if the women saw a skilled attendant compared with an unskilled attendant for ANC. In the total sample, women had higher odds of having an HIV test as part of their ANC if they went to a skilled attendant compared with if they went to an unskilled attendant (aOR: 1.78: 1.45–2.18). This increase in the odds of having an HIV test when utilizing a skilled attendant instead of an unskilled attendant for ANC was also demonstrated in Nigeria (aOR: 1.95: 1.30–2.93), Mozambique (aOR: 1.58: 1.22–2.05) and Uganda (aOR: 4.48: 2.48–8.08). Results from Congo did not demonstrate increased odds of having a HIV test as part of ANC if the women went to a skilled birth attendant compared with those who went to an unskilled birth attendant. Almost all Congolese women reported attending a skilled birth attendant for ANC (96.9%); however, less than half reported having a HIV test during ANC (45.5%). This is particularly troublesome as this is a missed opportunity for HIV education and entry into PMTCT care cascade.

Overall, the findings of our study are consistent with the literature on access to ANC services and uptake of HIV testing among pregnant women in low-income countries. For example, in a community-based, cross-sectional survey of mothers in Western Kenya, Kohler *et al*. [28] found that stigma and lack of HIV knowledge were significantly associated with a decreased likelihood of maternal HIV testing. Another study in rural Kenya [29] also established that women who demonstrated higher HIV-related stigma (i.e. those who held negative attitudes about persons living with HIV) were less likely to deliver in a health facility with a skilled attendant. As the authors explained based on their qualitative data, childbirth at a health facility was commonly viewed as most appropriate for women with pregnancy complications (e.g. HIV); therefore, women who deliver at health facilities may be labelled as HIV-positive in the community [29]. Age, education and residence have also been associated with being offered HIV counselling or testing, with younger and more educated women and those residing in urban settings more likely to be offered HIV counselling and testing than older, less-educated and rural women [30]. It was not surprising that women who receive their ANC services from unskilled health workers were less likely to receive HIV testing compared with those who received their service from skilled workers, because unskilled workers may not have access to HIV testing resources.

The findings from this study have implications for policy and practice regarding PMTCT of HIV in sub-Saharan Africa. They underscore the need for PMTCT and MCH programmes to pay particular attention to socioeconomic determinants of access to HIV testing as part of ANC services. Evidence from this study and several others [31–34] has revealed that women who lack comprehensive knowledge about HIV demonstrate stigmatizing attitudes towards persons living with HIV, have low educational levels, live in rural settings with difficult access to reproductive health services and receive their ANC services from unskilled workers are less likely to receive HIV testing as part of their ANC. This underscores the need for public health programmes in high HIV burden low-income countries to consider focusing their meagre resources on targeted efforts to reach pregnant women of these socioeconomic backgrounds. MCH programmes in sub-Saharan Africa should invest in efforts to reduce HIV stigma among pregnant women since stigma remains an important barrier to the uptake of HIV testing. Interventions that use peer-educators [35,36] as well culturally appropriate channels, such as faith-based institutions, can be effective not only in reducing stigma-related barriers but also geographical barriers [36]. Similarly, integrated testing approaches where HIV testing is considered as one of many tests related to maternal health, instead of HIV-only testing, can be effective in reducing the stigma and anxiety that are often associated with HIV testing [37,38]. Although it has been decades since the discovery of HIV/AIDS, there remain significant misconceptions about the disease. With the availability of ART at a relatively low direct cost to recipients in many low-income countries, the notion of HIV/AIDS as a death sentence should be dispelled by MCH programmes. The transmission of HIV from mother-to-child during pregnancy, labour, delivery or breastfeeding can be as high as 45% in the absence of interventions, but with early diagnosis, prompt initiation of care and adherence to PMTCT services, the rate can be reduced to less than 5% [39,40]. Early diagnosis and prompt enrolment into care also helps to preserve the health of the pregnant woman while reducing the chances of HIV transmission to a male partner who is not infected [41,42].

Since it is known that provider-initiated modes of testing make testing accessible to women from lower socioeconomic groups, ANC services should promote HIV counselling and testing as an essential component of ANC service for all pregnant women. Since many women, especially low-income women in rural settings, still rely mostly on unskilled workers for their antenatal services, MCH programmes should invest in efforts to train and integrate such unskilled workers into the formal ANC delivery system [43,44] and to provide them with access to HIV testing resources. With proper training and supervision, they can also be effective channels for dissemination of factual information about HIV and the benefits of testing and treatment and for referral to available treatment services [45,46].

The DHS data used for this study are not without limitations. The data from Congo, Mozambique and Uganda were collected in 2011–2012 and that from Nigeria in 2013. It is not known how well these data represent the current relationship between HIV testing and ANC care in their corresponding countries or in sub-Saharan Africa. It is also not known if there were any policy changes or events that may have affected the cohort in Nigeria in the two years after data were collected in the other three countries. Furthermore, there are several important questions that the DHS does not assess, including whether women knew their HIV status or were already on ART prior to ANC, how many ANC visits women had overall or in the first or second trimesters or how early HIV testing was performed. While these questions are beyond the scope of the DHS, they are important to know when considering the implementation and success of PMTCT services. Answering these questions would be an important future direction for empirical inquiry.

## Conclusions

This paper reveals individual and health system factors that influence women's uptake of HIV counselling and testing as part of their ANC services in sub-Saharan Africa. At the individual level, age, poverty, lack of HIV knowledge, stigma towards persons living with HIV/AIDS and low educational levels were associated with reduced uptake of HIV testing as part of ANC services. At the health system level, difficulty in accessing a health facility, rural residence and receipt of ANC services from unskilled attendants were associated with low HIV testing as part of ANC services. These findings have clear implications for the organization and delivery of ANC and PMTCT services in low- and middle-income countries (LMICs). As is often the case with vertical programmes in LMICs, ANC services may not be seen as routine but only necessary when something is amiss. Thus, health education may be necessary to change cultural norms and reduce missed opportunities for HIV testing of pregnant women. Outreach services may also be necessary as a strategy for reaching women in rural and difficult-to-reach settings. The value of such outreach services in MCH programming has been amply demonstrated in the literature [47,48]. Typically, in order to reduce adverse MCH outcomes, it is recommended that pregnant women receive their ANC services under the supervision of skilled attendants [49]. However, for cultural, geographical and economic reasons, the reality remains that a significant proportion of women in LMICs have limited access to either health facilities or skilled attendants. This underscores the need for ANC outreach programmes in these settings to identify unskilled attendants who provide ANC services to women in their jurisdiction so that they can be trained, integrated into the formal ANC delivery system, and regularly supervised [50]. Their propensity to provide pregnant women with HIV/AIDS education, refer them for testing and support those found to be HIV-positive through the PMTCT cascade of care could be enhanced through an incentive mechanism that rewards them per client referred to ANC or PMTCT HIV screening centres [51]. An alternative possibility is to provide unskilled ANC workers with access to HIV testing materials along with appropriate training (e.g. on testing procedures, result interpretation, maintenance of client confidentiality) and supervision. Given the association between knowledge of HIV, AIDS-related stigma and HIV testing, efforts should be directed at community education and sensitization about HIV and the importance of knowing one's status. The notion that HIV is a death sentence still prevails, especially in rural settings, and continues to create a barrier against the utilization of available services [28]. Education about HIV/AIDS in communities should focus on providing basic, factual information and on reducing prevailing misconceptions. The fact that treatment is available to help prevent transmission of HIV to children born to HIV-positive women should also be emphasized.

## Competing interests

None.

## Authors' contributions

JG, KC, IA and JE conceptualized the paper. IA and PW conducted data analyses, JG, KC, SG and JE drafted the manuscript. IA and EE commented and edited drafts of the manuscript. All authors reviewed and approved the manuscript for submission.
